# The Role of Nutrition on the Pathogenesis of Endometriosis

**DOI:** 10.3390/nu18040646

**Published:** 2026-02-16

**Authors:** Dominika Osińska, Andrzej Woźniak, Sławomir Woźniak

**Affiliations:** 1 The Independent Public Hospital No. 4 in Lublin, Jaczewskiego 8, 20-954 Lublin, Poland; 2 Department of Gynecology, Medical University of Lublin, Jaczewskiego 8, 20-954 Lublin, Poland

**Keywords:** endometriosis, nutrition, micronutrients, oxidative stress, inflammation, dietary patterns, curcumin

## Abstract

Background: Endometriosis is a chronic inflammatory gynecological disease affecting approximately 10% of women of reproductive age and is associated with pelvic pain, infertility, and reduced quality of life. Increasing evidence suggests that diet may influence endometriosis development and symptom severity through modulation of inflammation, oxidative stress, and hormone metabolism. This scoping review aimed to map current evidence on the role of nutrition in endometriosis. Methods: This scoping review was conducted in accordance with PRISMA-ScR guidelines. PubMed and Scopus were searched for original human studies published in English between 2014 and 2024. Eligible studies examined dietary patterns, food groups, micronutrients, bioactive compounds, body mass index, or fat consumption in relation to endometriosis risk, progression, or symptoms. Results: Diets rich in fruits and vegetables, including Mediterranean and low-FODMAP dietary patterns, were associated with reduced pain symptoms and improved quality of life. Antioxidant and anti-inflammatory nutrients, particularly vitamins C and D, zinc, and curcumin, were linked to modulation of oxidative stress, inflammation, angiogenesis, and cellular proliferation. Evidence regarding dairy intake, gluten, carbohydrates, dietary fat, and BMI was inconsistent or limited. Considerable heterogeneity across study designs and outcome measures was observed. Conclusions: Dietary factors may contribute to the modulation of endometriosis-related symptoms and underlying pathogenic mechanisms. Nutrients with antioxidant and anti-inflammatory properties appear promising as complementary, non-invasive strategies; however, methodological heterogeneity highlights the need for high-quality randomized controlled trials.

## 1. Introduction

Endometriosis is a chronic inflammatory gynecological condition characterized by the ectopic presence of active endometrial tissue outside the uterine cavity. Endometrial lesions may be located within the pelvic cavity, on the surface of the intestines, or in distant organs such as the lungs or the respiratory tract [1].

The American Society for Reproductive Medicine (ASRM) classifies endometriosis into four stages of severity: minimal, mild, moderate, and severe. The evaluation criteria include the number and depth of lesions, as well as the presence of endometrial cysts and adhesions. The disease progression is determined using a scoring system, which quantifies the extent and nature of the pathological alterations [2]. ASRM classification is widely used by scientists; however, it does not include the detailed anatomical location of the lesions, which makes it less useful for surgeons. Yet, the Enzian system uses anatomical compartments to describe where and how severely endometriosis is affecting pelvic organs and structures. Furthermore, it focuses on deep infiltrating endometriosis (DIE), which makes it more suitable for guiding surgical approach, technique, and calculating the risk of intraoperative complications [3].

The precise mechanisms underlying the pathogenesis of endometriosis remain incompletely understood; however, numerous hypotheses regarding its etiology have been proposed in the scientific literature. One of the most widely accepted theories is the concept of retrograde menstruation, which posits that blood containing viable endometrial cells retrogradely flows through the fallopian tubes into the peritoneal cavity, where it implants and proliferates [4]. Furthermore, studies indicate a significant association between the formation of endometrial implants and conditions of hyperestrogenism. The presence of ectopic endometrial tissue is also linked to localized inflammatory responses, which may lead to increased fibrosis and enhanced angiogenesis [5].

Endometriosis is a condition that affects approximately 170 million women worldwide, with its prevalence in the reproductive-age female population estimated at 10%. Among patients struggling with infertility, the disease is present in 21–47% of cases [6]. Characteristic symptoms of endometriosis include dysmenorrhea, dyspareunia, dysuria, and difficulties with conception [7]. Endometriosis also has a significant impact on the psychological well-being of patients, leading to the development of anxiety and depressive symptoms, as well as a decline in the quality of social relationships, which can result in feelings of exclusion and isolation [1].

The progression of endometriosis is influenced by a variety of factors, including anatomical, genetic, immunological, hormonal, and environmental conditions, with diet being one of the key environmental contributors [8]. Dietary habits can affect the development and progression of endometriosis through the regulation of steroid hormone metabolism, modulation of inflammatory responses, and management of oxidative stress [9]. Research suggests that increased intake of specific foods, such as fish oil, leafy green vegetables, and fresh fruits, may be associated with a reduced risk of endometriosis [10,11,12]. Conversely, diets high in processed meats and red meat, particularly beef, have been linked to a higher risk of developing the condition [13].

However, it is important to note that the current body of research examining the relationship between diet and endometriosis, both in terms of its onset and progression, remains limited. Consequently, further studies are urgently needed to gain a deeper understanding of the mechanisms underlying these associations.

## 2. Methodology

**Figure 1:**
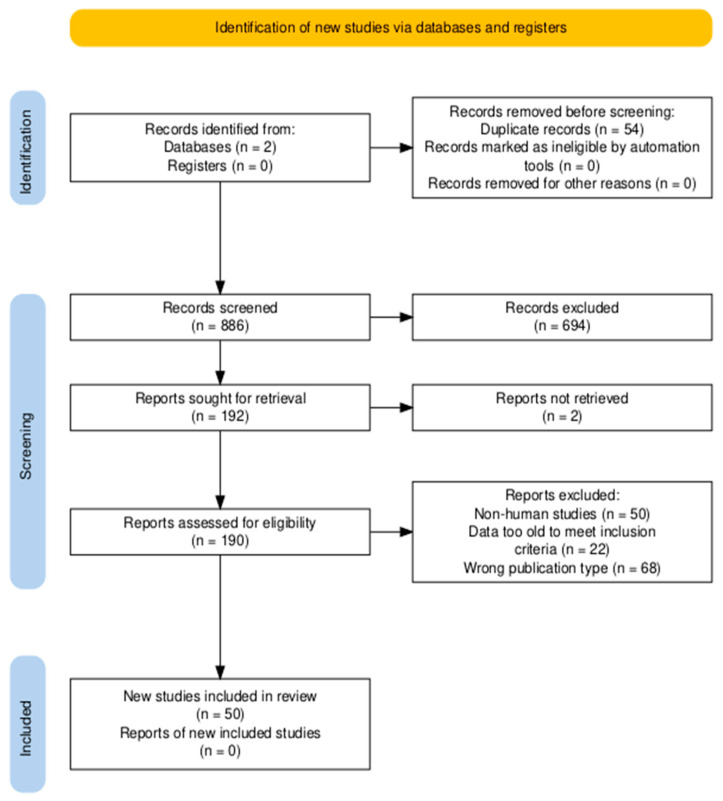
Identification of new studies via databases and registers.

The review was conducted in accordance with the PRISMA-ScR (Preferred Reporting Items for Systematic Reviews and Meta-Analyses extension for Scoping Reviews) guidelines [14]. The objective of this scoping review was to map and synthesize the available literature on the influence of dietary factors—including dietary patterns, food groups, micronutrients, bioactive compounds, body mass index, and fat consumption—on endometriosis risk, progression, and symptom severity. The articles were selected based on the inclusion criteria: publication type—original primary studies (observational, experimental studies) focusing on the influence of the diet on endometriosis; language of the study—English; and publication date ranging from 2014 to 2024. The time frame of the last ten years was chosen due to the rapid development of knowledge and increasing scientific interest in endometriosis and nutritional factors. Exclusion criteria were the publication type—review, meta-analysis, case report, and studies conducted on animals or on animal-derived cell lines. Specific details of excluded studies were not recorded. Research was conducted with Scopus and PubMed databases on 20 December 2024. The following search terms were used: “endometriosis diet”, “endometriosis vitamin C”, “endomeriosis vitamin D”, “endometriosis vitamin B”, “endometriosis vitamin A”, “endometriosis vitamin E”, “endometriosis fruit”, “endometriosis vegetables”, “endometriosis dairy”, “endometriosis carbohydrates”, “endometriosis zinc”, “endometriosis gluten”, “endometriosis fat”, “endometriosis olive oil”, “endometriosis micronutrient”, “endometriosis wheat”, “endometriosis curcumin”, and “endometriosis soy”. Search strategies were adapted to the specific requirements and syntax of each database. Searches were limited to English-language publications and applied to titles, abstracts, and keywords where applicable. The study selection process is presented using a PRISMA flow diagram (Figure 1). After duplicate removal, the remaining records were screened for eligibility using titles and abstracts and then full texts. The screening process was conducted by one reviewer. In cases of uncertainty regarding eligibility, a second reviewer was consulted to resolve discrepancies. No dedicated screening software or automation tools were used.

Data was organized using a standard extraction sheet in the Excel program. The extracted data included author(s), year of publication, study title, study design, number of participants, age group, type of dietary intervention or exposure (if applicable), main findings, and additional notes. Data extraction was performed by one reviewer.

The extracted data were synthesized using a descriptive and narrative approach. Studies were grouped and mapped according to the type of dietary component investigated (e.g., vitamins, micronutrients, food groups, or specific dietary compounds). The synthesis aimed to summarize research trends, identify knowledge gaps, and provide an overview of the existing evidence rather than to quantitatively pool results.

No formal assessment of the methodological quality or risk of bias of the included studies was performed, as this review was designed as a scoping review.

The review protocol was not registered. Given that this review was exclusively based on analysis of the publicly available literature and did not involve primary research involving human participants, it was exempt from Institutional Review Board (IRB) approval. This research received no external funding.

### 2.1. Vegetables and Fruits

Fruits and vegetables are key components of a balanced diet. Dietary patterns, particularly the intake of fruits and vegetables, have garnered increasing attention in the context of endometriosis [15].

Current research shows improvement of endometriosis pain symptoms and quality of life due to dietary interventions, such as following a low-FODMAP diet, “endometriosis diet”, or “fertility diet”, which are diets rich in vegetables and fruit [16,17]. A prospective one-center pilot study published in 2023, conducted on 62 participants, showed significant improvement in women’s well-being. Compared to the control group, women applying the low-FODMAP diet (low fermentable oligo-, di-, mono-saccharides and polyols diet), and the endometriosis diet reported less bloating and better scores in six out of eleven QoL (Quality of Life) domains after a 6-month period [18]. Another observational study, whose aim was to explore the effects of self-administered dietary modifications undertaken by Dutch women with endometriosis, also emphasizes the role of vegetable and fruit intake. According to the study results, women consuming more fruits and vegetables were more likely to report improvement in pain symptoms associated with endometriosis [19].

The Mediterranean diet (MD) is a dietary pattern traditionally followed in countries bordering the Mediterranean Sea. It is characterized by high consumption of vegetables, fruits, legumes, nuts, whole grains, and olive oil as the main source of fat; moderate intake of fish and poultry; low intake of red meat, processed foods, and sweets; and optional moderate consumption of dairy products and wine. This dietary pattern has been associated with anti-inflammatory and antioxidant effects. Research shows that it can significantly reduce oxidative stress levels, which are positively correlated to a higher perception of pain in women with endometriosis. According to the pre–post intervention study performed by Michela Cirillo et al. [20], Mediterranean dietary patterns can be useful for treating endometriosis-related symptoms in terms of dyspareunia, non-menstrual pelvic pain, dysuria, and dyschezia.

Current research from 2024 involving *Mahkota dewa* (*Phaleria macrocarpa*) demonstrates that the plant contains numerous therapeutically relevant compounds potentially beneficial for the treatment of infertility, which is frequently reported as a comorbid symptom of endometriosis [21]. The study demonstrated that the fruit ethanol extract contains bioactive compounds, including flavonoids, alkaloids, terpenoids, and steroids. Among these, flavonoids and steroids are particularly associated with fertility-related effects. Flavonoids have antioxidant, antiproliferative, anti-inflammatory, and antimutagenic effects, which can inhibit the proliferation of ectopic endometrium and reduce inflammation associated with endometriosis [22]. Steroids, also contained in the extract, are believed to modulate folliculogenesis, which may be relevant in the context of fertility. This study may serve as a basis for further research, especially in the context of the lack of non-invasive treatment options for endometriosis.

### 2.2. Dairy

The existing literature concerning the impact of dietary dairy intake on the risk of developing endometriosis and the severity of its symptoms remains limited and inconsistent. Certain studies suggest that higher dairy consumption may correlate with a reduced risk of endometriosis, whereas others report conflicting outcomes. A 2020 study involving 156 women in Tehran, Iran [23], indicated that dairy products contain phytoestrogens—such as isoflavones, lignans, and coumestrol—which were inversely associated with endometriosis risk. Conversely, a 2021 study [19] found that dietary modifications involving reduced dairy intake were linked to improvements in pain-related symptoms among women diagnosed with the disease. These divergent findings highlight the complexity of the relationship between dairy-derived nutrients and the pathophysiology of endometriosis.

### 2.3. Wheat, Carbohydrates, and Gluten

Current research on the role of gluten (wheat) and carbohydrates in endometriosis remains limited and inconsistent. Current research utilizing a Cox regression model reported a statistically insignificant association between dietary gluten intake and endometriosis risk [24]. However, another study demonstrated that among varied dietary approaches, removing gluten yielded preeminent results in decreasing the severity of endometriosis-related symptoms [19]. Moreover, no studies were identified that directly examined the impact of carbohydrate consumption on the development or progression of endometriosis. Given the ubiquity of carbohydrates and gluten in the human diet, there is a clear need for further investigation to elucidate their potential role in the progression of endometriosis.

### 2.4. BMI and Fat Consumption

There is a paucity of research examining the association between body mass index (BMI), fat consumption, and the risk of developing endometriosis. A 2022 study conducted in the United Kingdom [25] found that the relationship between high BMI and the onset of endometriosis is not linear. The findings indicated that having a body size larger than average at the age of 10 was associated with a higher prevalence of endometriosis. Interestingly, lower body weight in early childhood was also linked to an increased incidence of the disease. Nonetheless, other studies have demonstrated that both low BMI and a low waist-to-hip ratio (WHR) are associated with elevated risk of endometriosis [26,27], suggesting a complex and multifactorial relationship between these anthropometric measures and disease onset.

In addition, research focusing on dietary fat consumption has indicated that supplementation with olive oil or omega-3 polyunsaturated fatty acids may reduce pain symptoms and improve the quality of life in women with endometriosis. However, the observed trend did not reach statistical significance [28].

### 2.5. Vitamin B Group

Research regarding the influence of vitamin B on both the pathogenesis and progression of endometriosis is limited and inconsistent. A 2023 case–control study demonstrated a statistically significant decrease in the risk of developing the disease associated with increased dietary intake of vitamins B6, B2, and B12 [29]. Nonetheless, other studies have reported that high levels of vitamin B6 were positively correlated with a higher prevalence of endometriosis [30,31]. These findings indicate that the role of vitamin B in endometriosis remains unclear and underscore the need for further research.

### 2.6. Vitamin C

The body of research regarding the role of vitamin C in the diet of women with endometriosis is limited but consistent. Current evidence suggests that dietary supplementation with vitamin C, particularly in combination with vitamin E, can help reduce the severity of endometriosis symptoms, such as pelvic pain, dysmenorrhea, and dyspareunia. This effect is due to the reduction in vascular endothelial growth factor (VEGF), malondialdehyde (MDA), and reactive oxygen species (ROS) levels in the blood, which are strongly linked to oxidative stress and angiogenesis. Furthermore, research has shown that the total antioxidant capacity of the system was not diminished following treatment [32,33]. Another study demonstrated that vitamin C, in combination with other dietary micronutrients, also plays an important role in directly reducing the risk of developing the disease [29].

### 2.7. Vitamin D

Current evidence indicates that vitamin D can modulate inflammation and proliferation of the endometriotic cells through various processes [34]. Numerous studies show that vitamin D deficiency may increase the risk of developing endometriosis and contribute to its progression [35,36,37,38,39]. The evidence demonstrated that vitamin D can downregulate genetic pathways related to neuroangiogenesis and cellular invasion, which is critical for the pathogenesis of endometriosis [40]. Moreover, vitamin D may regulate excessive shedding of CD44 and attenuate β-catenin activity in endometrial stromal cells, which is crucial for cellular adhesion, migration, and proliferation [41,42]. Vitamin D is associated with a decrease in the expression of MCP-1, HGF, and IGF-1 proteins in blood and endometrial stromal cells, which are substantial for cell development and inflammation [43]. Furthermore, available evidence suggested a significant correlation between vitamin D intake, stage of endometriosis, and endometriosis-related symptoms, such as dysmenorrhea or pelvic pain [44,45]. Although few studies contradict the meaningful relationship between vitamin D intake and symptom improvement in women with endometriosis, they represent a small minority within the broader body of evidence [46,47].

### 2.8. Vitamin A

The existing literature pertaining to the role of vitamin A in the pathogenesis or progression of endometriosis is limited. A 2018 study conducted in Iraq found that diminished blood levels of antioxidant vitamins have a significant influence on ovarian steroid hormone production. Presented results indicated that decreased vitamin A levels, as well as increased oxidative stress markers, may enhance the activity of CYP19. The gene encodes aromatase, which is the key enzyme in estrogen biosynthesis. Polymorphism of the CYP19 gene was associated with increased prevalence of endometriosis [48]. Another study showed that substances chemically comparable to retinoic acid, such as transcrocetin, which is an apocarotenoid, may exhibit similar activity. The study suggested that transcrocetin can modulate inflammatory processes in endometriotic tissue by decreasing levels of proinflammatory cytokines, including IL-6 and MCP-1 [49].

### 2.9. Other Micronutrients

Micronutrients play a crucial role in disease development, which has led the current corpus of evidence to focus on their contribution to the prevalence of endometriosis.

Available evidence suggests that serum zinc levels in women with endometriosis were decreased [50]. Moreover, another study conducted in Ukraine on women treated in accordance with the Guideline Development Group recommendations showed that patients who received additional supplements consisting of zinc, resveratrol, and superoxide dismutase experienced significant relief in the case of endometriosis-related pain symptoms. This can indicate that zinc, in addition to other components of the supplement, may contribute meaningfully to decreasing oxidative stress, which is implicated in developing pain [51,52].

Current research has also demonstrated an inverse relationship between calcium and potassium intake and the risk of developing endometriosis [29]. This supports the notion that responsible intake of those micronutrients can actually help manage the symptoms. Yet another study showed that high levels of calcium, zinc, copper, and cobalt in women were linked to a higher prevalence of endometriosis [30,31]. However, those studies represented a small minority.

Additionally, the existing literature indicates a positive correlation between a low-nickel diet and improvement of gastrointestinal, extra-intestinal, and gynecological symptoms, which are associated with endometriosis or IBS. It may be due to the high frequency of allergic contact dermatitis, caused by alimentary nickel, in women with endometriosis. A 2020 study, conducted on 83 participants, showed that women reported a significant reduction in IBS-like symptoms after following a low-nickel diet for three months [53]. That may constitute the basis for further research on the role of nickel in indicating inflammation in endometriotic implants.

### 2.10. Curcumin

There is substantial evidence supporting the effects of curcumin on the pathogenesis of endometriosis. A considerable number of studies suggest its significant influence on oxidative stress, immunopathology, and cell proliferation processes. Current research has shown that curcumin can decrease the expression of TNFα in ectopic endometriotic cells, thus reducing inflammation. Additionally, curcumin affects the NF-κB signaling pathway by inhibiting the phosphorylation of NF-κB, subsequently suppressing the expression of proinflammatory cytokines [54,55]. The existing literature also indicates that curcumin downregulates the expression of vascular endothelial growth factor, consequently reducing the survival of human endometriotic stromal cells [56]. Furthermore, the anti-inflammatory and antiproliferative properties of curcumin, particularly when combined with other compounds, have been demonstrated to be highly effective in managing pain symptoms and preventing recurrence of ectopic lesions following surgery in patients with endometriosis [57,58,59,60]. Moreover, scientific findings support the notion that curcumin exhibits antioxidant effects. A 2022 study conducted on endometrial cells cultured from human endometriotic lesions demonstrated that copper–curcumin and nickel–curcumin complexes effectively accumulate in endometriotic cells and enhance both antioxidant activity and selective cytotoxicity [61]. Despite one report failing to demonstrate a beneficial effect, the overwhelming number of studies presenting positive outcomes suggests a robust and well-substantiated therapeutic role for curcumin [62].

### 2.11. Diet Influencing Pelvic Pain

It should be noted that most available studies assess multiple pain-related outcomes simultaneously, and therefore, the same studies are cited across symptom-specific sections. Available evidence specifically addressing dietary interventions targeting pelvic pain in endometriosis remains limited. However, several dietary components discussed earlier in this review have been consistently associated with a reduction in pelvic pain intensity. Diets rich in fruits and vegetables, such as the Mediterranean diet and low-FODMAP diet, have demonstrated beneficial effects, likely mediated through their antioxidant and anti-inflammatory properties [16,17,18,19,20]. Additionally, supplementation with omega-3 polyunsaturated fatty acids, olive oil, and vitamins C and D has been linked to reduced oxidative stress and inflammatory signaling, mechanisms strongly implicated in endometriosis-related pelvic pain [28,32,33,34,35,36,37,38,39,40,41,42,43,44,45].

### 2.12. Diet Influencing Dysmenorrhea

Evidence focusing specifically on dietary modulation of dysmenorrhea in endometriosis is scarce. Nonetheless, several micronutrients appear to influence menstrual pain severity. Vitamin C, particularly when combined with vitamin E, has been shown to reduce dysmenorrhea, potentially through the reduction in oxidative stress markers and angiogenic factors such as VEGF [32,33]. Moreover, vitamin D intake has been associated with decreased dysmenorrhea severity, possibly via modulation of inflammatory pathways and neuroangiogenesis involved in endometriotic lesion activity [44,45].

### 2.13. Diet Influencing Dyspareunia

Research investigating dietary interventions specifically targeting dyspareunia in endometriosis is limited. However, available studies suggest that adherence to dietary patterns with anti-inflammatory potential, such as the Mediterranean diet, may alleviate dyspareunia alongside other pain symptoms [20]. Similarly, antioxidant supplementation, including vitamins C and E, has been reported to reduce dyspareunia, likely by attenuating oxidative stress and inflammatory responses within endometriotic tissue [32,33]. These findings indicate that dyspareunia may improve indirectly through dietary modulation of systemic and local inflammation.

## 3. Strengths and Limitations of Available Studies

Studies analyzed in this review have several strengths. A substantial portion of research included women diagnosed with endometriosis via laparoscopy, which provides strong diagnostic validity. Moreover, a considerable amount of data regarding dietary behaviors was collected using validated Food Frequency Questionnaires (FFQs), ensuring standardized measurement. Most of the studies also adjusted for key confounders, such as BMI, age, or calorie intake. However, there are also some limitations. One of the main issues is the lack of blinding or controls in intervention studies. Furthermore, the existing heterogeneity in geographical diets, nutrients measured, and study endpoints can also limit the viability of the studies.

## 4. Conclusions

Available evidence indicates that increased consumption of fruits and vegetables may alleviate pain symptoms in patients with endometriosis, likely due to their antioxidant and anti-inflammatory properties. Additionally, emerging research on *Mahkota dewa* suggests that it may enhance reproductive function, attributed to its bioactive compounds, such as flavonoids and steroids.

Among vitamins, vitamin C—particularly when combined with vitamin E—has shown potential in reducing pain symptoms, possibly due to its antioxidative properties and its ability to decrease vascular endothelial growth factor (VEGF) levels, thereby limiting angiogenesis and reducing the proliferation of endometriotic implants. Consistent evidence regarding vitamin D suggests that it may attenuate key processes involved in the invasion of ectopic endometrial tissue, making it a promising adjunct therapy alongside conventional, often invasive, treatment approaches. Further research is needed to determine optimal dosing and long-term effects.

Other micronutrients have also demonstrated therapeutic potential: zinc appears to reduce oxidative stress, contributing to pain relief, while niacin (vitamin B3) has been associated with improvements in IBS-like symptoms often seen in endometriosis.

Findings related to dairy and gluten intake remain inconsistent, and the current evidence is insufficient to establish a clear correlation between fat consumption, BMI, and endometriosis symptoms.

Additionally, there is a growing body of research exploring the therapeutic potential of curcumin. Available evidence suggests that curcumin exhibits strong anti-inflammatory effects by decreasing the expression of pro-inflammatory cytokines in endometriotic cells. Moreover, it has been shown to reduce VEGF levels, thereby impairing angiogenesis and inducing apoptosis in ectopic lesions. Notably, curcumin has also been reported to prevent the recurrence of ectopic lesions following surgical intervention, highlighting its potential as an effective complementary therapy.

The evidence reviewed suggests that many endometriosis-related symptoms are closely linked to oxidative stress and chronic inflammation. Therefore, dietary components with antioxidant and anti-inflammatory properties may contribute to symptom alleviation. Additionally, substances that influence steroid hormone metabolism could be beneficial, given the association between endometriotic lesions and local hyperestrogenism.

This scoping review also highlights a substantial gap in the current literature. There is a clear need for more randomized controlled trials with standardized methodologies to strengthen the evidence base and guide clinical dietary recommendations for endometriosis management.

## Data Availability

No new data were created or analyzed in this study.

## References

[1] Rokitansky C. (1860). Über Uterusdrüsen-Neubildung in Uterus- und Ovarial-Sarcomen.

[2] Johnson N.P., Hummelshoj L., Adamson G.D., Keckstein J., Taylor H.S., Abrao M.S., Bush D., Kiesel L., Tamimi R., Sharpe-Timms K.L. (2017). World Endometriosis Society consensus on the classification of endometriosis. Hum. Reprod..

[3] Keckstein J., Saridogan E., Ulrich U.A., Sillem M., Oppelt P., Schweppe K.W., Krentel H., Janschek E., Exacoustos C., Malzoni M. (2021). The #Enzian classification: A comprehensive non-invasive and surgical description system for endometriosis. Acta Obstet. Gynecol. Scand..

[4] Burney R.O., Giudice L.C. (2012). Pathogenesis and Pathophysiology of Endometriosis. Fertil. Steril..

[5] Szubert M., Ziętara M., Suzin J. (2016). Czy istnieje możliwość diagnostyki endometriozy z poziomu endometrium?. Curr. Gynecol. Oncol..

[6] Della Corte L., Di Filippo C., Gabrielli O., Reppuccia S., La Rosa V.L., Ragusa R., Fichera M., Commodari E., Bifulco G., Giampaolino P. (2020). The Burden of Endometriosis on Women’s Lifespan: A Narrative Overview on Quality of Life and Psychosocial Wellbeing. Int. J. Environ. Res. Public Health.

[7] Polskiego S.Z.E. (2012). Stanowisko Zespołu Ekspertów Polskiego Towarzystwa Ginekologicznego dotyczące diagnostyki i metod leczenia endometriozy. Ginekol. Pol..

[8] Oral E., Arici A. (1997). Pathogenesis of endometriosis. Obstet. Gynecol. Clin. N. Am..

[9] Missmer S.A., Chavarro J.E., Malspeis S., Bertone-Johnson E.R., Hornstein M.D., Spiegelman D., Barbieri R.L., Willett W.C., Hankinson S.E. (2010). A prospective study of dietary fat consumption and endometriosis risk. Hum. Reprod..

[10] Sakine G.S., Ghazaleh E., Neda K.S., Bahram R., Robabeh T., Elahe N. (2022). Association of dietary intakes and anthropometric indices with endometriosis: A case-control study. Iran. J. Obstet. Gynecol. Infertil..

[11] Ashrafi M., Jahangiri N., Sadatmahalleh S.J., Aliani F., Akhoond M. (2020). Diet and the Risk of Endometriosis in Iranian Women: A Case-Control Study. Int. J. Fertil. Steril..

[12] Youseflu S., Sadatmahalleh S.J., Mottaghi A., Kazemnejad A. (2019). The Association of Food Consumption and Nutrient intake with Endometriosis Risk in Iranian Women: A Case-Control Study. Int. J. Reprod. Biomed. (IJRM).

[13] Parazzini F., Chiaffarino F., Surace M., Chatenoud L., Cipriani S., Chiantera V., Benzi G., Fedele L. (2004). Selected food intake and risk of endometriosis. Hum. Reprod..

[14] Tricco A.C., Lillie E., Zarin W., O’Brien K.K., Colquhoun H., Levac D., Moher D., Peters M.D.J., Horsley T., Weeks L. (2018). PRISMA Extension for Scoping Reviews (PRISMA-ScR): Checklist and Explanation. Ann. Intern. Med..

[15] Morales-Prieto D.M., Herrmann J., Osterwald H., Kochhar P.S., Schleussner E., Markert U.R., Oettel M. (2018). Comparison of dienogest effects upon 3,3′-diindolylmethane supplementation in models of endometriosis and clinical cases. Reprod. Biol..

[16] van Haaps A., Wijbers J., Schreurs A., Mijatovic V. (2023). A better quality of life could be achieved by applying the endometriosis diet: A cross-sectional study in Dutch endometriosis patients. Reprod. Biomed. Online.

[17] Ghasemisedaghat S., Eslamian G., Kazemi S.N., Rashidkhani B., Taheripanah R. (2023). Association of fertility diet score with endometriosis: A case–control study. Front. Nutr..

[18] van Haaps A.P., Wijbers J.V., Schreurs A.M.F., Vlek S., Tuynman J., De Bie B., de Vogel A.L., van Wely M., Mijatovic V. (2023). The effect of dietary interventions on pain and quality of life in women diagnosed with endometriosis: A prospective study with control group. Hum. Reprod..

[19] Krabbenborg I., de Roos N., van der Grinten P., Nap A. (2021). Diet quality and perceived effects of dietary changes in Dutch endometriosis patients: An observational study. Reprod. Biomed. Online.

[20] Cirillo M., Argento F.R., Becatti M., Fiorillo C., Coccia M.E., Fatini C. (2023). Mediterranean Diet and Oxidative Stress: A Relationship with Pain Perception in Endometriosis. Int. J. Mol. Sci..

[21] Brahmana I.B., Purwanto P., Soetrisno S., Purwanto B., Pamungkasari E.P. (2024). Qualitative and Quantitative Tests on the Ingredients of *Mahkota dewa* (*Phaleria macrocarpa*) Fruit Extract as an Alternative Therapy for Infertility in Endometriosis. Res. J. Pharm. Technol..

[22] Youseflu S., Sadatmahalleh S.J., Mottaghi A., Kazemnejad A. (2019). Evaluation of the role of dietary flavonoid intake in the risk of endometriosis among Iranian women. Iran. J. Obstet. Gynecol. Infertil..

[23] Youseflu S., Sadatmahalleh S.J., Mottaghi A., Kazemnejad A. (2020). Dietary Phytoestrogen Intake and the Risk of Endometriosis in Iranian Women: A Case-Control Study. Int. J. Fertil. Steril..

[24] Eliyati N., Maiyanti S., Dwipurwani O., Hamidah S. (2021). Model Regresi Cox Untuk Menganalisis Pengaruh Faktor Asupan Makanan Terhadap Risiko Kekambuhan Endometriosis. BAREKENG J. Ilmu Mat. Dan Terap..

[25] Venkatesh S.S., Ferreira T., Benonisdottir S., Rahmioglu N., Becker C.M., Granne I., Zondervan K.T., Holmes M.V., Lindgren C.M., Wittemans L.B.L. (2022). Obesity and risk of female reproductive conditions: A Mendelian randomisation study. PLoS Med..

[26] Hanifah A.V., Budihastuti U.R., Balgis B. (2022). The Association Between Body Mass Index, Waist to Hip Ratio and Mid-Upper Arm Circumference with Endometriosis. Unnes J. Public Health.

[27] da Silva C.V.D., Felipe V.L., Shivappa N., Hebert J.R., Perini J.A., de Brito P.D., Cardoso J.V., Ferrari R., Filho G.L.d.A. (2021). Dietary Inflammatory Index score and risk of developing endometriosis: A case-control study. J. Endometr. Pelvic Pain Disord..

[28] Abokhrais I.M., Denison F.C., Whitaker L.H.R., Saunders P.T.K., Doust A., Williams L.J., Horne A.W. (2020). A two-arm parallel double-blind randomised controlled pilot trial of the efficacy of Omega-3 polyunsaturated fatty acids for the treatment of women with endometriosis-associated pain (PurFECT1). PLoS ONE.

[29] Roshanzadeh G., Jahanian Sadatmahalleh S., Moini A., Mottaghi A., Rostami F. (2023). The relationship between dietary micronutrients and endometriosis: A case-control study. Int. J. Reprod. Biomed..

[30] Zhang Y., Li M., Zhang F., Lin J., Yuan H., Nian Q. (2024). Circulating micronutrients levels and their association with the risk of endometriosis. Front. Nutr..

[31] Su X., Yue X., Zhang Y., Shen L., Zhang H., Wang X., Yin T., Zhang H., Peng J., Wang X. (2024). Elevated levels of Zn, Cu and Co are associated with an increased risk of endometriosis: Results from a case control study. Ecotoxicol. Environ. Saf..

[32] Amini L., Chekini R., Nateghi M.R., Haghani H., Jamialahmadi T., Sathyapalan T., Sahebkar A. (2021). The Effect of Combined Vitamin C and Vitamin E Supplementation on Oxidative Stress Markers in Women with Endometriosis: A Randomized, Triple-Blind Placebo-Controlled Clinical Trial. Pain Res. Manag..

[33] Ansariniya H., Hadinedoushan H., Javaheri A., Zare F. (2019). Vitamin C and E supplementation effects on secretory and molecular aspects of vascular endothelial growth factor derived from peritoneal fluids of patients with endometriosis. J. Obstet. Gynaecol..

[34] Miyashita M., Koga K., Izumi G., Sue F., Makabe T., Taguchi A., Nagai M., Urata Y., Takamura M., Harada M. (2016). Effects of 1,25-Dihydroxy Vitamin D3 on Endometriosis. J. Clin. Endocrinol. Metab..

[35] Chu T.-W., Jhao J.-Y., Lin T.-J., Lin T.-W., Wang C.-L., Chang H.-S., Liu L.-C., Chang C.-C. (2021). Vitamin D in gynecological diseases. J. Chin. Med. Assoc..

[36] Delbandi A.-A., Torab M., Abdollahi E., Khodaverdi S., Rokhgireh S., Moradi Z., Heidari S., Mohammadi T. (2021). Vitamin D deficiency as a risk factor for endometriosis in Iranian women. J. Reprod. Immunol..

[37] Yarmolinskaya M., Denisova A., Tkachenko N., Ivashenko T., Bespalova O., Tolibova G., Tral T. (2021). Vitamin D significance in pathogenesis of endometriosis. Gynecol. Endocrinol..

[38] Denisova A.S., Yarmolinskaya M.I., Tkachenko N.N. (2021). Assessment of 25(OH)D status in patients with genital endometriosis and clinical efficacy of cholecalciferol in the treatment of the disease. J. Obstet. Women’s Dis..

[39] Ciavattini A., Serri M., Delli Carpini G., Morini S., Clemente N. (2017). Ovarian endometriosis and vitamin D serum levels. Gynecol. Endocrinol..

[40] Ingles S.A., Wu L., Liu B.T., Chen Y., Wang C.-Y., Templeman C., Brueggmann D. (2017). Differential gene expression by 1,25(OH)2D3 in an endometriosis stromal cell line. J. Steroid Biochem. Mol. Biol..

[41] Pazhohan A., Amidi F., Akbari-Asbagh F., Seyedrezazadeh E., Aftabi Y., Abdolalizadeh J., Khodarahmian M., Khanlarkhani N., Sobhani A. (2018). Expression and shedding of CD44 in the endometrium of women with endometriosis and modulating effects of vitamin D: A randomized exploratory trial. J. Steroid Biochem. Mol. Biol..

[42] Pazhohan A., Danaei-Mehrabad S., Mohamad-Rezaeii Z., Amidi F., Khodarahmian M., Shabani Nashtaei M., Sobhani A., Farajzadeh M.A. (2021). The modulating effects of vitamin D on the activity of β-catenin in the endometrium of women with endometriosis: A randomized exploratory trial. Gynecol. Endocrinol..

[43] Heidari S., Kolahdouz-Mohammadi R., Khodaverdi S., Mohammadi T., Delbandi A.-A. (2022). Changes in MCP-1, HGF, and IGF-1 expression in endometrial stromal cells, PBMCs, and PFMCs of endometriotic women following 1,25(OH)2D3 treatment. J. Cell. Mol. Med..

[44] Abadi A., Rizany S., Agustiansyah P., Nurtjahyo A., Irfanuddin Usman F., Hartati Mirani P., Krisna R., Amran R., Prasetiyo M.A.T. (2024). Serum Vitamin D Levels, Visual Analog Scale Dysmenorrhea Score, and Endometriosis ASRM Classification: A Relationship Study. Indones. J. Obstet. Gynecol..

[45] Mehdizadehkashi A., Rokhgireh S., Tahermanesh K., Eslahi N., Minaeian S., Samimi M. (2021). The effect of vitamin D supplementation on clinical symptoms and metabolic profiles in patients with endometriosis. Gynecol. Endocrinol..

[46] Nodler J.L., DiVasta A.D., Vitonis A.F., Karevicius S., Malsch M., Sarda V., Fadayomi A., Harris H.R., Missmer S.A. (2020). Supplementation with vitamin D or ω-3 fatty acids in adolescent girls and young women with endometriosis (SAGE): A double-blind, randomized, placebo-controlled trial. Am. J. Clin. Nutr..

[47] Almassinokiani F., Khodaverdi S., Solaymani-Dodaran M., Akbari P., Pazouki A. (2016). Effects of Vitamin D on Endometriosis-Related Pain: A Double-Blind Clinical Trial. Med. Sci. Monit..

[48] Al-Rubae’I S.H., Naji T.S., Turki K.M., Edan D.S. (2018). Association of the G/T rs4646 of CYP19 gene polymorphism with oxidative stress, vitamin A and estradiol in Iraqi women with endometriosis disease. Gene Rep..

[49] Ham J., Song J., Song G., Lim W. (2024). Autophagy regulation and redox perturbation by transcrocetin suppress the growth of endometriosis. Biomed. Pharmacother..

[50] Messalli E., Schettino M., Mainini G., Ercolano S., Fuschillo G., Falcone F., Esposito E., Di Donna M., De Franciscis P., Torella M. (2014). The possible role of zinc in the etiopathogenesis of endometriosis. Clin. Exp. Obstet. Gynecol..

[51] Semenyna H., Hrytsko M., Doroshenko-Kravchyk M., Korytko O., Fartushok T. (2024). New opportunities for correction of hormonal disorders and oxidative stress in women with genital endometriosis. Int. J. Endocrinol..

[52] Rabajdová M., Špaková I., Smolko L., Abrahamovská M., Baranovičová B., Birková A., Vašková J., Mareková M. (2024). Serum trace element levels and activity of enzymes associated with oxidative stress in endometriosis and endometrial cancer. FEBS Open Bio.

[53] Borghini R., Porpora M.G., Casale R., Marino M., Palmieri E., Greco N., Donato G., Picarelli A. (2020). Irritable Bowel Syndrome-Like Disorders in Endometriosis: Prevalence of Nickel Sensitivity and Effects of a Low-Nickel Diet. An Open-Label Pilot Study. Nutrients.

[54] Banerjee S., Xu W., Doctor A., Driss A., Nezhat C., Sidell N., Taylor R.N., Thompson W.E., Chowdhury I. (2023). TNFα-Induced Altered miRNA Expression Links to NF-κB Signaling Pathway in Endometriosis. Inflammation.

[55] Chowdhury I., Banerjee S., Driss A., Xu W., Mehrabi S., Nezhat C., Sidell N., Taylor R.N., Thompson W.E. (2019). Curcumin attenuates proangiogenic and proinflammatory factors in human eutopic endometrial stromal cells through the NF-κB signaling pathway. J. Cell. Physiol..

[56] Cao H., Wei Y.-X., Zhou Q., Zhang Y., Guo X.-P., Zhang J. (2017). Inhibitory effect of curcumin in human Endometriosis endometrial cells via downregulation of Vascular endothelial growth factor. Mol. Med. Rep..

[57] Fadin M., Nicoletti M.C., Pellizzato M., Accardi M., Baietti M.G., Fratter A. (2020). Effectiveness of the integration of quercetin, turmeric, and *N*-acetylcysteine in reducing inflammation and pain associated with endometriosis. In-vitro and in-vivo studies. Minerva Ginecol..

[58] Signorile P.G., Viceconte R., Baldi A. (2018). Novel dietary supplement association reduces symptoms in endometriosis patients. J. Cell. Physiol..

[59] Zakharenko N., Regeda S., Manoliak I., Solskyy V. (2021). Anti-relapse therapy of endometriosis: Possible variations. Reprod. Endocrinol..

[60] Zupi E., Lazzeri L., Centini G. (2015). Endometriosis and pain: Postsurgical alternative treatment in patents desiring pregnancy. J. Endometr..

[61] Singh A., Ghosh P., Mukherjee S., Ojha A.K., Hansda A., Choudhury P., Halder S., Sharma S., Mukherjee G., Dasgupta S. (2022). Transition metallo-curcumin complexes: A new hope for endometriosis?. J. Mater. Chem. B.

[62] Gudarzi R., Shabani F., Mohammad-Alizadeh-Charandabi S., Naghshineh E., Shaseb E., Mirghafourvand M. (2024). Effect of curcumin on painful symptoms of endometriosis: A triple-blind randomized controlled trial. Phytother. Res..

